# Address

**Published:** 1879-09

**Authors:** C. N. Pierce


// THE DENTAL REGISTER.

VOL. XXXIII.] SEPTEMBER, 1879. No. 9

### ADDRESS.

BY PROF. C. N. PIERCE.

*Read before the Pennsylvania State Dental Society.*

### DENTISTRY—ITS OPPORTUNITIES AND OBLIGATIONS.

Life and its responsibilities, bring with them, domestic, civil, and religious duties that can not be overlooked or ignored. Every man is indebted to society, not only for his inherited, but his acquired culture, and for this, society claims in return a compensating influence; for his having lived, it should be made better. Not less is the individual obligation to a profession. Its years of probation and growth have given it position, its accumulated culture has given it influence, and any one entering it is benefited in proportion to his ability to appropriate these results and advantages. By choice of a profession an obligation is assumed, of which one should ever be mindful, and this stimulating influence should create a desire to contribute something for the benefit of his associates. Membership, and attendance upon monthly as well as these annual gatherings, is not without a recognized educational and truly beneficial influence. Intercourse of man with man, mind with mind, stimulates activity, provokes thought, elevates desires, leads to new and better methods, and excites to novel inventions, so that thereby we are continually acquiring new power, peacefully annexing to our own domain new provinces of nature, multiplying labor-saving appliances, and setting wind and water, fire and lightning to do our work, and, with the aid of these, saving an amount of time which should be spent in some ennobling pursuit.

How to employ advantageously this liberated time is an important inquiry; that it should be so occupied as to promote a desirable culture is doubtless true, but so differently do minds develop, and so varied is the amount of force which nature puts at our disposal that an accurate estimate of our tastes and capacities becomes an important factor in our progress. "We must determine upon the relative values of knowledge," and to do this we must properly estimate the requirements of our life work, and accurately measure our relative deficiencies. This again involves the inquiry as to the extent or limitations of our occupation, and whether our studies in this or that direction will contribute most to our professional efficiency and success.

### THE CONNECTION OF ANATOMY AND PHYSIOLOGY WITH THE PROFESSIONAL STUDY OF DENTISTRY.

Concerned as I am for the elevation of the profession, to which I have given twenty-seven years of active interest, I must beg your indulgence for a few minutes, while I present the claims of the anatomical and physiological studies, which hold so intimate a relation to our duties, that to call them collateral, gives them a place more foreign than is warranted. Anatomy, "the science of the structure of living tissues," opens a field so broad and important, that in its general outlines it should be understood by every parent and teacher, but when studied as special, microscopical or comparative anatomy, its interest and importance is so intensified that life seems too short to compass its almost unlimited field. Comparative anatomy has not only familiarized us with the typical mammalian tooth, of which those of the human species are, doubtless, but modifications, but it offers us the best explanation for the differentiation of tooth forms, by pointing out the various mandibular movements and showing that, co-existent with these, is a peculiar type of tooth formation, and "that, as these excursive movements of the jaw have increased in complexity, there has been an apparent increase in the complexity of the enamel foldings, ridges and crests."

It has also shown us that while the typical number of teeth in the mammalia was forty-four, this has been gradually reduced by the modification of food and use to the thirty-two, which with their imperfections are common to the human species, and which seem prophetic in the course of centuries of an almost edentulous jaw. It has demonstrated beyond a doubt that the difference in the size of the human jaws, and in the size and structure of the human teeth and their attachments, are but modifications in correspondence with the difference in use, and again it has demonstrated that this is largely due to the changes or modifications in food from the solid to the soft, from the uncooked to the cooked.

Physiology, embracing as it does biology, in introducing us to that department of natural science which treats of the "laws, processes, functions and phenomena of life," as well as the study of vital force and nutrition, leads us back to the very verge of inorganic matter. Vitality is the first condition of animal existence, a condition determining growth and maintenance, renewal and regeneration, in the incipient as well as in the more mature stages of organisms. In the contemplation of this we are carried to the commencement of life, the morphological unit, the primary and fundamental forms of life, an individual mass of protoplasm, in which no other or further structure is discernible. We may follow this mass through its first process of development by division and subdivision into a number of morphological units, and with these build up the cell.

THE LAW OF INHERITED FORMS.

"Behold, now, a fecundated cell," so small and apparently so homogeneous that neither chemistry nor microscopy can detect its parentage, and yet in that ovum reside physical, mental and moral, potentialities, peculiarities of class, order, species, family, individuality. In that ovum is every living germ; "there is the assured promise of all the organs which, in their harmony and co-ordination, make a perfect organism." There is the tendency to reproduce the character of its immediate parents. No inherited structural modification so slight, no functional peculiarity so insignificant, no moral obliquity so monstrous in either parent that it may not be wrapped up in this little cell, abiding its allotted time to make its appearance in the offspring.

From this microscopic germ we trace the various progressive steps from the simple to the complex, and note the gradual differentiation of organs and functions, recognizing the same developmental process that is observable in the progress from the lower to the higher organisms; and just here we note the fact that "physiologically as well as morphologically development is a progress from the general to the special."

Before leaving this subject, let me again refer to that most remarkable and inexplicable study of heredity—the imperfections in structure, and the predispositions to disease that are transmitted from one or both parents to the offspring. No occupation affords a better opportunity for observing these than does ours. What organs are more readily impressed by systemic conditions than the teeth? What structures more certainly modified in quantity or quality than the dental?

Upon the teeth of thousands of children is written in letters of gold the sins of their fathers. Many a pure wife suffers the humiliation of seeing her first-born disfigured for life through the licentiousness of her husband. What dentist has not recognized with unerring precision the cause of the peculiar malformation of the teeth of this or that child? or, as he has watched and treated with care some case of maxillary necrosis, fully realized that the sins of the parent are surely visited upon the children?

### THE MICROSCOPE IN DENTISTRY.

Not less allied to our profession than the foregoing studies of anatomy and physiology is that of microscopy; with the aid of its little spheres, multiplying world upon world, we are afforded such golden opportunities for self-instruction! Of the interest it adds to our professional labors we may well be proud, revealing to us, as it does, a differentiation of tissue and a wonderfully delicate and vascular structure which to the unaided vision is in appearance naught but a homogeneous, structureless mass. Of the improved instruments of research which have aided so much to the sciences embraced in the healing art, there is no one which takes such unchallenged precedence in the assistance it has rendered to the anatomist, physiologist, histologist and pathologist, as does the microscope. Previous to its use nothing was definitely known respecting the development, organization or structure of tissue, and, until its penetrating eye revealed the true appearance of tissue in health, the changes made by the ravages of disease were unknown to the pathologist. As students, in our examination of tissues, normal or morbid, whose strucures are too minute to be accurately seen without artificial assistance, this instrument of precision is of incalculable advantage to us.

In no branch of anatomy have its revelations added greater wealth of knowledge, or given more definite information, than in studying the development and structure of the teeth. From the unfolding of the epithelial layer, and on through its various progressive changes, with the modifications in the sub-strata of cells for the dental and cemental structures, and their preparation for the disposition of the salts of lime, all is so beautifully and wonderfully unfolded by the revelations of this instrument, that what once was wrapped in mystery and viewed with superstition, is now lined out with mathematical precision from its inception to its completion, so that, through the instrumentality of this piece of mechanism, mystery yields to intelligence and superstition, to wonder and admiration, as we study and appreciate the wisdom displayed in the law governing the development of such structures.

I have thus briefly sketched these allied studies, that we may the more clearly realize the scope of our professional life, and fully appreciate the opportunities it offers for culture. Truly the harvest is abundant and rich in the fullness of the grain.

Let us not then lament the narrowness of our field, or complain of the limited demands of our occupation, while subjects of such interest are mastered by so few, but rather let us labor, and labor unceasingly, remembering that "He that soweth shall reap, and whosoever hath to him shall be given, and he shall have more abundantly."

I have spoken thus of these three branches not only because of the near relation they hold to our special industry, but because I believe most emphatically that such knowledge widens our opportunity for good; nay more than this, I believe that as our professional labors have provided for our physical wants, and have insured the needful comforts for those dependent upon us, a still deeper, wider, and not less interesting and important field is opened before us, by pursuing further these or other branches of the natural sciences. It is not enough that our handiwork should have erected untarnished monuments of our skill, or our appreciative patients should hold us in pleasant memory for the services we have rendered; for all of this we have received a commensurate compensation. Let us, then, give a portion of our leisure moments to the more thorough study of some one branch of science, and in this way add something to the stature of our profession for the revenue it has yielded, and for the position it has made possible. Believing, as I do, in the elevating and ennobling tendency of the study of natural sciences, I think we can not pursue any department or branch "without being imbued with a love of Nature as she displays herself in the ever-varying attitude of organized existences;" and though our selection of a subject should carry our interest and sympathies beyond the confines of our daily life, we should be broadened and benefited by the "truth discovered anywhere within the physico-vital records of a past and passing world."

Accurate and enlarged conceptions of the structural, morphological and functional peculiarities and their modifications, exhibited by the multitudinous organisms which nature unfolds, have a wonderful influence in training the powers of observation, and, if not over-balanced by some unfavorable conditions, exercises a most salutary effect in quieting the manner and preventing brag and pretension. Culture kills conceit and exaggeration, and at the same time advances social and intellectual worth. In confirmation of this let me quote the following from an address delivered some years since by Prof. Huxley, "On Natural History, as Knowledge, Discipline and Power." He says:

"Let those who doubt the efficacy of science as moral and mental discipline, make the experiment of trying to come to a comprehension of the meanest worm or weed, of its structure, its habits, its relation to the great scheme of nature. It will be a most exceptional case if the mere endeavor to give a correct outline of its form, or describe its appearance with accuracy, does not call into exercise far more patience, perseverance and self-denial than they have easily at command; and if they do not rise up from the attempt in utter astonishment at the habitual laxity and inaccuracy of their mental processes, and in some dismay at the pernicious manner in which their subjective conceptions and hasty preconceived notions interfere with their forming a truthful comprehension of the objective fact. There is not one person in fifty whose habits of mind are sufficiently accurate to enable him to give a truthful description of the exterior of a rose."

### THE VALUE OF SPECIAL LINES OF RESEARCH.

I have spoken of education, of the opportunities offered in our profession of the elevating influence of the study of the natural sciences, A word or two more respecting the methods of study, and I shall not tax you longer. It is not the superficial knowledge of a number of branches that counts for education, or makes the man a source of wealth, in society, but the thorough mastery of one; so let us not waste our strength in attempting too much, but remember that from the general to the special is the march of progress.

"The art of enthusiasm" and concentration of force is what every man who makes himself authority in any department must learn.

Espy's labors, night after night, upon his housetop watching the formation, movements and dissolution of clouds culminated in our daily weather reports.

Agassiz and Tyndall, summer after summer at Chamounix, gave us the movements of the glacier of the Aar. The studies of Rosa Bonheur among the cattle, Hinckley with the dogs, Landseer with the deer, add beauty to our homes, and make these animals familiar companions of city life.

Leidy and Cope in Paleontology; Le Coute and Horn in Entomology; Kollicker, Leydig and Haeckel in Histology; Audubon and Gould in Ornithology; Hunt in Microscopy; Tryon in Conchology, with a host of others, each pursuing his individual specialty with such industry, enthusiasm and pride, that an undoubted confidence is placed in the published results of their researches. This same division of labor is seen in the science of geology, mineralogy, botany, chemistry and physics, each worker in his favorite science, "considering his own the flower, and perfect consummation of the intellectual world." This is the beauty and strength of the science of the present century; "harmony in diversity, multiplicity in unity."

It is the untiring labor and zeal of such men as Spencer, Darwin, Haeckel and Huxley, which has brought so prominently before the people the theory of evolution as against that of emanation, and whether or not subsequent revelations sustain it in its fullest proportions, it is a matter of the greatest congratulation that the indomitable courage and industry of these men and their co-laborers have done so much to relieve society of ignorance and superstition.

In estimating the value of study and education, let us not think the end and object of it all is the accumulation of gold or personal aggrandizement, but rather to brighten the face and lessen the burden of life, to strengthen in the human heart contentment with its lot, to increase that faith which believes "that all things are well ordered and sure, and work together for good."

